# Wide diagnostic and genotypic spectrum in patients with suspected mitochondrial disease

**DOI:** 10.1186/s13023-023-02921-0

**Published:** 2023-10-02

**Authors:** Kristina Grigalionienė, Birutė Burnytė, Laima Ambrozaitytė, Algirdas Utkus

**Affiliations:** Department of Human and Medical Genetics, Institute of Biomedical Sciences, Faculty of Medicine, Vilnius University, Santariškių Str. 2, Vilnius, LT-08661 Lithuania

**Keywords:** Mitochondrial disease, mtDNA, nDNA, Genetic diagnosis, Variants, Next generation sequencing, Whole exome sequencing

## Abstract

**Background:**

Mitochondrial Diseases (MDs) are a diverse group of neurometabolic disorders characterized by impaired mitochondrial oxidative phosphorylation and caused by pathogenic variants in more than 400 genes. The implementation of next-generation sequencing (NGS) technologies helps to increase the understanding of molecular basis and diagnostic yield of these conditions. The purpose of the study was to investigate diagnostic and genotypic spectrum in patients with suspected MD. The comprehensive analysis of mtDNA variants using Sanger sequencing was performed in the group of 83 unrelated individuals with clinically suspected mitochondrial disease. Additionally, targeted next generation sequencing or whole exome sequencing (WES) was performed for 30 patients of the study group.

**Results:**

The overall diagnostic rate was 21.7% for the patients with suspected MD, increasing to 36.7% in the group of patients where NGS methods were applied. Mitochondrial disease was confirmed in 11 patients (13.3%), including few classical mitochondrial syndromes (MELAS, MERRF, Leigh and Kearns-Sayre syndrome) caused by pathogenic mtDNA variants (8.4%) and MDs caused by pathogenic variants in five nDNA genes. Other neuromuscular diseases caused by pathogenic variants in seven nDNA genes, were confirmed in seven patients (23.3%).

**Conclusion:**

The wide spectrum of identified rare mitochondrial or neurodevelopmental diseases proves that MD suspected patients would mostly benefit from an extensive genetic profiling allowing rapid diagnostics and improving the care of these patients.

**Supplementary Information:**

The online version contains supplementary material available at 10.1186/s13023-023-02921-0.

## Background

Mitochondrial diseases (MDs) are a clinically heterogeneous group of genetic disorders caused by defects affecting the function of the mitochondrial respiratory chain and mitochondrial oxidative phosphorylation pathway. The estimated collective prevalence of MDs in adults is approximately 1 in 4,300 (1 in 11,500 in children) [1].

MD may occur at any age and may involve any organ and tissue, most commonly affecting organs that require high energy levels, such as the nervous system, heart, eyes, skeletal muscles, liver, and kidney. This results in a diversity of clinical presentations and a wide spectrum of clinical symptoms including psychomotor delay or regression, epilepsy, cerebellar ataxia, encephalopathy and stroke-like episodes, dystonia, myopathy, exercise intolerance, sensorineural deafness, ophthalmoplegia, ptosis, optic atrophy, pigmentary retinopathy, cardiomyopathy, diabetes mellitus, etc. [1–3] Most MDs are progressive and have poor prognosis with high morbidity and mortality. Although management and treatment is limited to supportive care for the vast majority of MDs, a growing portfolio of pharmacological and genetic therapies are in preclinical development or reaching clinical trials [4].

In addition to the clinical heterogeneity, the genetics of MDs is extremely complex, as it is estimated that more than 1,500 mitochondrial proteins are involved in mitochondrial structure, biogenesis and proper respiratory chain functioning [5]. The majority of these proteins are encoded by nuclear DNA (nDNA) and are essential for mtDNA maintenance, mitochondrial dynamics, coenzyme Q10 biosynthesis, assembling of the respiratory chain complexes, activity and turnover, but 13 essential subunits of respiratory chain complexes are encoded by mitochondrial genome. Therefore, mitochondria are under the control of two genomes, and MDs result from pathogenic variants either in nDNA or in mtDNA [6]. 1136 human genes are currently linked to mitochondrial proteome and more than 400 genes are recognized as playing role in MDs following every inheritance pattern, but the list of candidate genes is still growing up [7].

In mtDNA, the variant may not be present in all of the multiple mtDNA copies per cell, the coexistence of wild-type and mutated mtDNA molecules is known as heteroplasmy. Mutant load of mtDNA may vary from tissue to tissue and over time, contributing to the highly variable disease expression. mtDNA variants are maternally inherited with unpredictable levels of heteroplasmy or can also be sporadic or secondary to nuclear genes’ mutations [8].

Given the considerable clinical and genetic heterogeneity associated with MDs, the diagnosis of MDs is really challenging. Some well-defined clinical syndromes are associated with specific mtDNA mutations, however, in the majority of cases, clinical manifestations are diverse, with overlap between conditions, making specific MDs difficult to identify and differentiate clinically. MD patients sharing the same genotype may have limited phenotypic congruence, and conversely, the similar phenotypes can be caused by different genetic mechanisms [9]. Moreover, other genetic and aquired diseases can lead to a secondary respiratory chain deficiency. Currently, there are no reliable biomarkers to diagnose MDs. Diagnosis is typically defined by a complex combination of clinical assessment, blood and cerebrospinal fluid metabolite profiles, brain imaging, tissue histology and enzymology, and specific nuclear gene and/or mtDNA sequencing [8, 10]. Some of those clinical criteria have been formalized into scoring systems [11–13]. Nevertheless, those diagnostic methods have been costly, invasive, time-consuming, and in many cases have failed to provide a molecular diagnosis. The identification of the responsible gene is a prerequisite for proper genetic counseling, prenatal or preimplantation genetic diagnosis and sometimes indicates treatment options [3, 6].

Genome-wide approaches including whole exome sequencing (WES) and whole genome sequencing (WGS) in complement with the transcriptome analysis by RNA sequencing (RNA-seq) have overcome the need to prioritize candidate genes for targeted analysis in MDs. Despite the great advantage of these approaches, the diagnostic success rate of massively parallel sequencing is still reliant on careful clinical and biochemical characterization of patients, and to date, more than one out of two patients has no diagnosis, as genetic defect remains unknown [3, 6, 8].

The aim of the present study was to investigate diagnostic and genotypic spectrum in a group of Lithuanian patients with suspected MDs. The study was performed during the process of reforming our diagnostic approach from traditional diagnostic methods to NGS-based diagnostics. Therefore, traditional diagnostic approaches, as well as NGS-based metods were used in the present study.

## Results

The study included 83 unrelated patients (50 females and 33 males) with suspected MD. All patients were evaluated and scored based on a modified Nijmegen disease severity score [11, 12]. The structure of the studied group according to the age at presentation of the clinical symptoms and mitochondrial disease criteria (MDC) scores is shown in Table 1.

**Table 1:** The structure of the study group

	No. of pediatric patients (< 16 years, n = 58)	No. of adult patients (> 16 years, n = 25)
Definite MD (MDC score 8–12)	8	4
Probable MD (MDC score 5–7)	14	11
Possible MD (MDC score 2–4)	36	10

mtDNA screening for large-scale rearrangements and complete mtDNA sequencing was performed for all patient samples. Nuclear mitochondrial genes were investigated using targeted gene panel sequencing or WES technology in 9 and 21 cases, respectively (30 cases in total accounting 36% of all patients).

In total, likely causal variants were identified in 18/83 cases (21.7%), conclusive molecular MD or other diagnosis was obtained in 17 of these cases (20.5%) and probable diagnosis in one case (1.2%). Five novel, sixteen known pathogenic or likely pathogenic variants and one known variant of uncertain significance were identified. The overall diagnostic rate according to used investigation methods, the age of disease onset, MDC scores, established disorder (mitochondrial or other disease) and genome changes (mtDNA or nDNA) is summarized in Table 2.

**Table 2:** Overall diagnostic rate of the study

	No. of cases	No. of solved cases	Diagnostic rate, %
**Testing method**			
mtDNA sequencing	80	6	**7.5**
mtDNA del/dup analysis	83	1	**1.2**
Targeted gene NGS (without further WES analysis)	9	3	**33.3**
WES	21	8	**38.1**
**The age of disease onset**			
Pediatric (< 16 years)	58	13	**22.4**
Adult (> 16 years)	25	5	**20.0**
**MDC scores**			
Definite MD (MDC score 8–12)	12	4	**33.3**
Probable MD (MDC score 5–7)	25	10	**40.0**
Possible MD (MDC score 2–4)	46	4	**8.7**
**Established disorder**			
Mitochondrial disease	83	11	**13.3**
Other disease (not related to primary mitochondrial dysfunction)	83	6	**7.2**
Both mitochondrial and other disease identified	83	1	**1.2**
**Genome change**			
mtDNA	83	7	**8.4**
nDNA	83	10	**12.0**
mtDNA + nDNA	83	1	**1.2**
**Total**	83	18	**21.7**

### Mitochondrial disease genes

Mitochondrial genome analysis confirmed MD diagnosis in eight patients. Six known pathogenic variants in *MT-TL1, MT-ATP6* and *MT-TK* causing Mitochondrial encephalomyopathy, lactic acidosis and stroke-like episodes (MELAS), Leigh and Myoclonus epilepsy associated with ragged-red fibers (MERRF) syndromes, respectively, were identified (Table 3). In addition, one patient was confirmed as carrying common pathogenic mtDNA variant causing Leber hereditary optic neuropathy (LHON) although the finding did not fit the clinical phenotype of the patient and NGS analysis was further performed to specify the diagnosis. Furthermore, mtDNA deletion / duplication analysis identified a novel single 5888 bp deletion involving 13 mtDNA genes and confirmed the clinical diagnosis of Kearns-Sayre syndrome (KSS) in one patient [14].

MD diagnosis was also confirmed in three (10.0%) of the 30 patients tested for pathogenic variants in nuclear genes. Pathogenic variants were detected in *TRMU*, *SURF1* and *PNPLA8* genes. One additional patient (3.3%) received a probable diagnosis with only one heterozygous pathogenic variant found in *RRM2B* and pathogenic variants in *BTD* gene. Although the second *RRM2B* variant was not identified for this patient, additional mtDNA quantification showed low mtDNA copy number in muscle biopsy (Table 3). In all cases, an autosomal recessive mode of inheritance was observed.

**Table 3:** Variants identified in mitochondrial disease genes in the patient group

Patient ID	Sex/ age of onset (years)	MDC score	Gene (RefSeq)	Nucleotide and Amino acid change	Clinical significance (met pathogenicity criteria according to ACMG)	Clinical phenotype (MIM#), inheritance	Testing method
**mtDNA variants**							
MT-020	F/>16	Definite (10)	*MT-TL1 *(NC_12920.1)	heteroplasmic variant: m.3243A>G (tRNA Leu)	Known pathogenic (PS3, PM2, PM5, PP3)	MELAS (540000), maternal	mtDNA sequencing
MT-028	F/>16	Probable (5)	*MT-TL1 *(NC_12920.1)	heteroplasmic variant: m.3243A>G (tRNA Leu)	Known pathogenic (PS3, PM2, PM5, PP3)	MELAS (540000), maternal	mtDNA sequencing
MT-041	F/>16	Definite (12)	*MT-TL1 *(NC_12920.1)	heteroplasmic variant: m.3243A>G (tRNA Leu)	Known pathogenic (PS3, PM2, PM5, PP3)	MELAS (540000), maternal	mtDNA sequencing
MT-058	M/<16	Probable (6)	*MT-ATP6 *(NC_12920.1)	heteroplasmic variant: m.8993T>C, p.(Leu156Pro)	Known pathogenic (PP5, PS3, PM5, PP3)	Leigh syndrome (256000), maternal	mtDNA sequencing
MT-049	F/<16	Probable (7)	*MT-ATP6 *(NC_12920.1)	heteroplasmic variant: m.9185T>C, p.(Leu220Pro)	Known pathogenic (PP5, PS3, PP3)	Leigh syndrome (256000), maternal	mtDNA sequencing
MT-086	F/>16	Probable (6)	*MT-TK *(NC_12920.1)	heteroplasmic variant: m.8344A>G (tRNA Lys)	Known pathogenic (PP5, PS3, PM2, PP3)	MERRF (545000), maternal	WES
MT-073	M/<16	Possible (2)	*MT-ND4 *(NC_012920.1)	homoplasmic* variant: m.11778G>A, p.(Arg340His)	Known pathogenic (PP5, PS3, PM5, PP3)	Leber optic atrophy (535000), maternal	mtDNA sequencing
MT-027	M/<16	Probable (7)	13 genes (NC_12920.1)	de novo* heteroplasmic variant: m.6068_11956del	Novel pathogenic (PVS1, PM2, PM6, PM1)	KSS (530000), sporadic	mtDNA del/dup analysis
**nDNA variants**							
MT-056	F/<16	Definite (8)	*SURF1* (NM_003172.4)	homozygous* variant: c.845_846del, p.(Ser282CysfsTer9)	Known pathogenic (PVS1, PP5, PM2)	Mitochondrial complex IV deficiency, nuclear type 1 (256000), AR	WES
MT-024	F/<16	Probable (7)	*TRMU* (NM_018006.5)	homozygous* variant: c.835G>A, p.(Val279Met),	Known likely pathogenic (PP5, PM2, BP1, BP6)	Liver failure, transient infantile (613070), AR	WES
MT-085	M/<16	Probable (6)	*PNPLA8 *(NM_001256007.3)	compound heterozygous* variants: 1. c.2269del, p.(Thr757GlnfsTer10) 2. c.2275_2276del, p.(Leu759AlafsTer4)	1. Novel pathogenic (PVS1, PM2, PM3) 2. Known likely pathogenic (PVS1, PM2, PM3, PP5)	Mitochondrial myopathy with lactic acidosis (251950), AR	WES
Patient with inconclusive diagnosis							
MT-042	M/<16	Definite (8)	*RRM2B* (NM_015713.5)	heterozygous variant: c.414_415del, p.(Tyr138Ter)	Known likely pathogenic (PVS1, PM2)	Mitochondrial DNA depletion syndrome 8 A (encephalomyopathic type with renal tubulopathy) or 8B (MNGIE type) (612075), AR	Targeted gene NGS
			*BTD* (NM_001370658.1)	compound heterozygous* variants: 1. c.583C>T, p.(Leu195Phe) 2. c.1270G>C, p.(Asp424His)	1. Known likely pathogenic (PM1, PP2, PM2, PP3, PM3, PP5) 2. Known pathogenic (PM1, PP2, PP3, PM3, PP5, BA1, BS2)	Partial biotinidase deficiency (253260), AR	Targeted gene NGS

* The *de novo*, homozygous or compound heterozygous origin of the variant was confirmed by testing the parients. Abbreviations: AD – autosomal dominant, AR – autosomal recessive, F – female, M – male, MDC – mitochondrial disease criteria, MELAS - Mitochondrial encephalomyopathy, lactic acidosis and stroke-like episodes, MERRF - Mitochondrial encephalomyopathy, lactic acidosis and stroke-like episodes, KSS - Kearns-Sayre syndrome, NGS – next generation sequencing, WES – whole exome sequencing

### Genes not obviously related to mitochondrial function

Seven patients of 83 received a confirmed genetic diagnosis of other diseases as pathogenic or likely pathogenic variants were identified in genes with no previous evidence of causing a MD. Three cases had dominant inheritance (two autosomal and X-linked), and 3 cases were autosomal recessive (one homozygous and two compound heterozygous). Limb-girdle muscular dystrophy was diagnosed in one patient after identifying two likely causal variants in *ANO5* gene, but common mitochondrial variant m.11778G > A associated with LHON was also identified (Table 4).

**Table 4:** Variants identified in genes not obviously related to mitochondrial function in the patient group

Patient ID	Sex/ age of onset (years)	MDC score	Gene (transcript)	Nucleotide and Amino acid change	Clinical significance (met pathogenicity criteria according to ACMG)	Clinical phenotype (MIM#), inheritance	Testing method
MT-050	F/<16	Possible (3)	*CACNA1A* (NM_001127222.2)	de novo* heterozygous variant: c.4043G>A, p.(Arg1348Gln)	Known pathogenic (PS2, PM1, PP2, PM2, PM5, PP3, PP5)	Developmental and epileptic encephalopathy 42 (617106), AD	Targeted gene NGS
MT-061	F/<16	Possible (3)	*DDX3X* (NM_001356.5)	de novo* heterozygous variant: c.1629_1630dup, p.(Phe544TyrfsTer8)	Novel pathogenic (PVS1, PM2, PM6)	Intellectual developmental disorder, X-linked, syndrome, Snijders Blok type (300958)	WES
MT-040	F/<16	Probable (6)	*TPP1* (NM_000391.4)	homozygous* variant: c.622C>T, p.(Arg208Ter)	Known pathogenic (PVS1, PM2, PP5)	Ceroid lipofuscinosis, neuronal, 2 (204500), AR	WES
MT-069	M/<16	Possible (4)	*KIF1A* (NM_001244008.2)	de novo* heterozygous variant: c.173C>T, p.(Ser58Leu)	Known pathogenic (PP3, PS2, PM2, PM5, PP2, PP5)	Autosomal dominant non-syndromic intellectual disability (ORPHA:178469), AD	WES
MT-029	M/<16	Probable (7)	*YARS1* (NM_003680.4)	compound heterozygous* variants: 1. c.176T>C, p.(Ile59Thr) 2. c.1440del, p.(Glu480AspfsTer32)	1. Known likely pathogenic (PM3, PM2, PP3, PP5) 2. Novel likely pathogenic (PM3, PM2, PVS1)	Infantile-onset multisystem neurologic, endocrine, and pancreatic disease 2 (619418), AR	WES
MT-073	M/<16	Possible (2)	*ANO5* (NM_213599.3)	compound heterozygous* variants: 1. c.1407+5G>A, p.(?) 2. c.2137A>G, p.(Thr713Ala)	1. Known pathogenic (PM2, PM3, PP3, PP5) 2. Known VUS (PM3, PM2)	Muscular dystrophy, limb-girdle, autosomal recessive 12 (611307), AR Miyoshi muscular dystrophy 3 (613319), AR	Targeted gene NGS
			*MT-ND4* (NC_012920.1)	homoplasmic variant: m.11778G>A, p.(Arg340His)	Known pathogenic (PP5, PS3, PM5, PP3)	Leber optic atrophy (535000), maternal	mtDNA sequencing
MT-054	M/<16	Probable (6)	*SETD5* (NM_001080517.3)	heterozygous variant: c.2347-2del, p.(?)	Novel likely pathogenic (PVS1, PM2)	Intellectual developmental disorder, autosomal dominant 23 (615761)	WES

* The homozygous or compound heterozygous origin of the variant was confirmed by testing the parients. Abbreviations: AD – autosomal dominant, AR – autosomal recessive, F – female, M – male, MDC – mitochondrial disease score, NGS – next generation sequencing, XL – X-linked, WES – whole exome sequencing

## Discussion

In past decade, rapid development of advanced technologies in genetics and genomics allowed to dramatically increase the identification of the genetic causes of mitochondrial disorders.

This study was performed during a period when traditional research methods were being replaced by NGS technologies, therefore different molecular genetic methods were applied to study a group of patients with clinically suspected MD. The genetic diagnosis was conclusive in 17 of 83 cases, inconclusive in 1 of 83 cases, giving the overall diagnostic rate of 21.7%. Unfortunately, for the rest of the patients the diagnosis still remains undetermined.

The present study allowed to identify pathogenic variants in either mtDNA or nDNA genes. Some of the diagnoses were clinically suspected and consistent with the phenotype. For example, a patient with clinically diagnosed Kearns-Sayre syndrome was found to have a *de novo* heteroplasmic single 5888 bp mtDNA deletion m.6069_11956del [14]. Common pathogenic mtDNA variants were identified as causing MELAS and Leigh syndromes. However, some of the findings expanded the knowledge of possible phenotypic presentation associated with some genes. For instance, *PNPLA8* gene encodes mitochondrial phospholipase iPLA2γ which is involved in mitochondrial membrane lipid metabolism and is required for efficient bioenergetic function [15]. Biallelic pathogenic variants in *PNPLA8* gene have been recently associated with a severe mitochondrial neurodegenerative disease in children, manifesting with microcephaly, hypotonia, weakness, epilepsy, global developmental delay, poor weight gain, and lactic acidosis [16, 17]. The patient reported in this study presented with milder phenotype consisting of adolescence onset ataxia and progressive sensorimotor polyneuropathy [18]. Thus, variability in the manifestation of *PNPLA8*-related disease seemingly exists.

In this study, one patient received a probable diagnosis with only one heterozygous pathogenic variant identified for autosomal recessive MD. We were able to identify only one previously reported [19] heterozygous pathogenic variant c.414_415del p.(Tyr138Ter) in *RRM2B* gene. The 2-month-old boy suffered from severe weakness, feeding difficulties, progressive deterioration of respiratory function, hearing loss, and generalized hypotonia with minimal spontaneous movements in legs and significantly reduced deep tendon reflexes. Lactate was elevated. A muscle biopsy showed 20% of ragged-red fibers, > 95% fibers negative for cytochrome-c oxidase, and abnormal deposits of lipids. The disease progressed rapidly and the boy died at the age of 3 months. Additionally, partial biotinidase deficiency caused by pathogenic variants in *BTD* gene was confirmed for the patient [20]. However, the patient’s phenotype was consistent with autosomal recessive infantile-onset RRM2B-related mitochondrial DNA depletion syndrome [21]. The second pathogenic variant in *RRM2B* was not identified, as pathogenic variants in deep introns or other regulatory sequences were not covered by targeted gene NGS. RNA sequencing approach could be the choice to further test the patient to identify variants causing aberrant splicing, aberrant or even monoallelic expression. For the patients with WES-inconclusive results, transcriptome study by RNA-seq has shown the increase of diagnostic yield by 10–16% for rare mitochondrial and other Mendelian diseases [22, 23].

Other disease was confirmed for seven patients. One patient showed multisystem involvement suggesting the presence of MD, yet, biallelic likely pathogenic variants were identified in *YARS1* gene. *YARS1* gene encodes tyrosyl-tRNA synthetase that catalyzes the aminoacylation of the amino acid tyrosine to its corresponding tRNA(Tyr) [24]. This activity is essential for translation and protein synthesis. Pathogenic variants in *YARS1* gene have been recently associated with infantile-onset multisystem neurologic, endocrine, and pancreatic disease. In other case, the male patient was referred due to ocular symptoms and diagnosed as a carrier of common familial mitochondrial DNA variant m.11778G>A resulting Leber hereditary optic neuropathy. Later he was referred with isolated hyperCKemia, further genetic analysis identified biallelic variants in *ANO5* gene. Several other neurodevelopmental disorders, manifesting with developmental delay, hypotonia, movement disorders, mild dysmorphic features were identified. Most of pathogenic variants identified in *CACNA1A*, *DDX3X*, *KIF1A*, and *SETD5* genes were *de novo* with autosomal or X-linked dominant inheritance pattern. The unspecific clinical and biochemical findings of those pediatric patients allowed the clinicians to consider these patients as having possible or even probable MD, but in individuals who do not present with a recognisable clinical syndrome, the suspicion of MD becomes part of the differential diagnosis of complex neurogenetic disorders. WES may be an indispensable tool to differentiate and reach the final diagnosis of those rare cases.

The limited diagnostic rate of the study can be explained by several reasons. In the study, mtDNA was tested in all patients, the identified diagnostic rate for point mtDNA variants using Sanger sequencing method was 7.5%. Such diagnostic rate could be explained by the fact that pathogenic variants in mtDNA account for only part of the causes of MDs. Moreover, the sensitivity of Sanger sequencing method is limited to detect pathogenic variants at low levels of heteroplasmy (e.g. if not appropriate tissue is tested), therefore some of the variants may remain undetected. mtDNA deletion / duplication analysis identified only one single large-scale deletion and confirmed diagnosis of Kearns-Sayre syndrome. Few patients with chronic progressive external ophthalmoplegia (CPEO) were included in the study, therefore, more positive results were expected. However, mtDNA deletions were not identified in those patients, and possible reason for that could be the tested tissue. It has been shown that deletions and duplications are often undetectable in blood samples in patients over 20 years of age [25], so other tissues tested may have been a better option in these patients.

Pathogenic variants in nuclear genes were tested in 30 patients of the study group, and the detection rate of 36.7% was estimated for NGS methods. The present data are comparable with other data reported in the literature, where a diagnostic rate of targeted gene panels for suspected MD is indicated as 7–31%, while WES achieved diagnostic yields of 25–70% [26, 27]. The identification of pathogenic variants may face limitations due to untested DNA regions, including limited gene panel lists or uncovered regulatory regions in WES. Technical limitations of NGS, such as insufficient coverage of certain regions, may also lead to missed variants. Additionaly, interpreting clinical significance of the identified variants and evaluating gene functions is challenging. Some variants, particularly those associated with a specific disease phenotype, are pathogenic, while others require segregation analysis. WES of family trios or functional studies may be more appropriate for analyzing variants of uncertain significance [28].

Studies show that higher diagnostic rates were obtained in groups with complete phenotyping and more accurate selection of patients with suspected MD [8, 19, 29]. In the study, the patients were categorized as having definite, probable, or possible MD score. Genetic diagnosis was established in only 8.7% of patients with possible MD, while the majority of diagnoses were conclusive in patients with probable (40.0%) and definite (33.3%) MD. A significant limitation of the current study was the small proportion of patients tested using NGS methods. The lower diagnostic rate in patients with definite MD compared to probable MD may be explained by the fact that only 3 of 12 (25%) patients with definite MD were tested using NGS methods compared to 12 of 25 (48%) patients with probable MD and 15 of 46 (33%) patients with possible MD. MD was confirmed mostly in patients classified as having definite or probable MD, while other diseases were diagnosed mostly in patients classified as having probable or possible MD. Patients classified as having probable MD had a high prevalence of clinical symptoms suggesting mitochondrial disease, even if later confirmed to have other disease. Our results showed that patients with higher MDC were more likely to receive not only MD diagnosis, but also an overall genetic diagnosis. It is likely that the scoring system classifies not only primary MD patients but also patients with some other neurodevelopmental diseases, presenting with overlapping clinical symptoms of MD or possible secondary mitochondrial dysfunction.

## Conclusions

In the study, the overall diagnostic rate was 21.7% for the patients with suspected MD, increasing to 36.7% in the group of patients where NGS techniques were applied. The data suggest that WES should be used as a first-choice genetic diagnostic method for the patients with multisystem, neurodevelopmental or neuromuscular manifestation, as many patients harbor disease causing variants in both MD genes and genes not obviously related to mitochondrial function, and overlapping clinical phenotypes might not always indicate MD.

## Methods

### Patients

Eighty three unrelated patients were included in the study: 58 pediatric (under 16 years of age) and 25 adult (over 16 years of age). All patients were evaluated and scored based on a modified mitochondrial disease criteria (MDC) scale [11, 12]. Although the Nijmegen mitochondrial disease criteria were developed in children with mitochondrial disease, they were used for our cohort as the clinical presentations included in these diagnostic criteria reflect the phenotypic variability. Most of the adults enrolled in the study had multi-systemic disorders (≥ 3 affected organs), characteristically affecting tissues with high-energy demands, such as central nervous system, skeletal muscle, eye, and heart. Some patients had a recognizable mitochondrial syndrome.

### Ethical considerations

DNA samples and data collection were performed in accordance to the Declaration of Helsinki, the study protocol was approved by the Vilnius Regional Biomedical Research Ethics Committee of Lithuania. Written informed consent was obtained from all patients or their parents before involvement in the study.

### Sample preparation

Molecular genetic testing for the patients was performed using total DNA extracted from peripheral blood samples via standard procedures using the phenol-chloroform-isoamyl alcohol extraction method. In few cases DNA samples extracted from urine epithelial cells or muscle biopsies were also available for the study.

### mtDNA deletion / duplication analysis

Long-range polymerase chain reactions (LR-PCR) using two different pairs of primers and multiplex ligation-dependent probe amplification (MLPA) method using Mitochondria Salsa MLPA Kit P125 (MRC-Holland, Amsterdam, The Netherlands) were applied for mtDNA deletion / duplication analysis in all patients. Detailed analysis protocols were previously published [14].

### mtDNA sequencing

For complete mitochondrial genome sequencing nine overlapping fragments of approximately 1700–3000 bp in length were amplified using Phusion Hot Start II DNA Polymerase according to the manufacturer’s protocol (Thermo Fisher Scientific, USA). Oligonucleotide sequences for PCR amplification are provided in Table S1 (Additional file 1). Each fragment was sequenced with 3–5 forward and 3–5 reverse primers. The sequences of the 62 oligonucleotide sequencing primers are provided in Table S2 (Additional file 1). Sanger sequencing was performed using the BigDye™ Terminator v3.1 Cycle Sequencing Kit (Applied Biosystems, USA), and automatic genetic analyser ABI PRISM 3130xl (Applied Biosystems, USA) according to the manufacturer’s protocol.

### nDNA panel sequencing

Targeted amplicon NGS for mitochondrial and other neuromuscular disorders was applied for 9 patients with suspected MD. The library preparation was performed using an Ion AmpliSeq Library Kit 2.0 and Ion AmpliSeq™ Neurological Research Panel consisting of 752 genes associated with neurological disorders and including 99 genes related to mitochondrial diseases (Thermo Fisher Scientific, USA; Table S3 (Additional file 1)). Enrichment of exonic sequences was performed with an Ion PGM™ Hi-Q™ View OT2 Kit (Thermo Fisher Scientific, USA) on the Ion OneTouch™ 2 System (Life Technologies, Thermo Fisher Scientific, USA) and sequenced on an Ion PGM™ System (Life Technologies, Thermo Fisher Scientific, USA) using Ion PGM™ Hi-Q™ View Sequencing Kit (Thermo Fisher Scientific, USA) according to the manufacturer’s protocol. Mapping and variants calling were performed using the Ion Torrent Suite™ Server v. 5.0.2 (Thermo Fisher Scientific, USA).

### Whole-exome sequencing

Whole exome sequencing (WES) was performed for 21 patients. Primary steps of raw sequencing data i.e. demultiplexing and trimming of the adaptors were performed by the subcontracting NGS provider (CeGaT GmbH, Tübingen, Germany) using high-throughput next-generation Illumina (Illumina, Inc., San Diego, CA, USA) platform. Obtained sequencing data (FASTQ, BAM, VCF files) analysis was further performed in our laboratory using validated in-house bioinformatic pipeline.

### Bioinformatic analysis of sequence variants

#### mtDNA variants

mtDNA sequencing data obtained using Sanger sequencing were analysed using BioEdit v.7.2 The obtained sequences were compared with the revised Cambridge Reference Sequence (rCRS, GenBank number NC_012920.1).

All mtDNA variants obtained using WES were filtered out. mtDNA sequencing read depth in WES data was on average 400–600. mtDNA variants present at lower than 20% heteroplasmy level were not detected using standard analysis algorithm. The GenBank NC_012920.1 reference sequence was used for analysis and reporting of mtDNA variants.

Homoplasmic and heteroplasmic variants were evaluated on the basis of their allele frequency according to Mitomap Frequency, gnomAD 3.1 Frequency and Helix Frequency provided in MITOMAP database [30]. Variants with a population allele frequency < 0.1% were considered significant (with some exceptional known pathogenic variants present in populations at higher frequencies). Variant pathogenicity was evaluated according to criteria proposed for mtDNA variants [31–33]. The pathogenicity of mtDNA variants was evaluated using MITOMAP, HmtVar manually curated database [34], NCBI ClinVar [35], Varsome [36] databases as well as scientific literature. The pathogenicity of mitochondrial tRNA variants was evaluated using MitoTIP *in silico* prediction algorithm [37]. APOGEE [38] meta-predictor and other common *in silico* tools (SIFT [39], PolyPhen-2 [40], etc.) were used to predict pathogenicity of the variants in protein‐coding genes.

#### Nuclear DNA variants

Annotation and post-annotation of obtained sequencing data (FASTQ, BAM, VCF files) from both, panel and WES sequencing, were further post-processed on site using validated in-house bioinformatic pipeline (including ANNOVAR [41]). Reads were mapped/aligned to the Human NCBI Build GRCh37 (hg19/2009) reference genome, duplicated reads marked before variant calling and annotation. In WES data, CNVs were annotated separately from SNP/indels using AnnotSV program. Prioritization and interpretation of genome variants were performed using genomic tools and databases provided by ANNOVAR program in up to ± 6 bp of flanking regions and up to 2% minor allele frequency (MAF). Criteria provided by the American College of Medical Genetics and Genomics (ACMG) and the Association for Molecular Pathology (AMP) [42], *in silico* tools and databases (e.g., SIFT, PolyPhen-2, GERP++ [43], CADD [44], ExAC [45], GnomAD [46], 1000 Genome Project data [47], NCBI dbSNP [48], NCBI ClinVar, Varsome, etc.), and the relevant literature were used to assess the pathogenicity of detected variants. Clinically significant variants were reported following HGVS recommendations [49].

### Electronic supplementary material

Below is the link to the electronic supplementary material.

**Additional file 1: Table S1**. Oligonucleotide sequences for PCR amplification of mtDNA. **Table S2**. Oligonucleotide sequencing primers for mtDNA Sanger sequencing. **Table S3**. A list of 751 targeted genes associated with mitochondrial and other neuromuscular disorders tested using targeted gene next generation sequencing technology (Ion AmpliSeq™ Neurological Research Panel). MD-associated genes (99) are bolded

## Data Availability

The technical data supporting the findings of this study are available within the paper and its Supplementary Information. Oligonucleotide sequences for PCR amplification are provided in Table S1 (Additional file 1). The sequences of the oligonucleotide sequencing primers are provided in Table S2 (Additional file 1). The list of genes tested using targeted amplicon NGS (Ion AmpliSeq™ Neurological Research Panel) is provided in Table S3 (Additional file 1). Clinical and genetic data that support the findings of this study are not openly available due to reasons of sensitivity and are available from the corresponding author upon reasonable request.

## Abbreviations

AD: Autosomal dominant
AR: Autosomal recessive
CPEO: Chronic progressive external ophthalmoplegia
DNA: Deoxyribonucleic acid
F: Female
KSS: Kearns-Sayre syndrome
LHON: Leber hereditary optic neuropathy
M: Male
MD: Mitochondrial disease
MDC: Mitochondrial disease criteria
MELAS: Mitochondrial encephalomyopathy, lactic acidosis and stroke-like episodes
MERRF: Myoclonus epilepsy associated with ragged-red fibers
mtDNA: Mitochondrial DNA
nDNA: Nuclear DNA
NGS: Next-generation sequencing
RNA-seq: RNA sequencing
RNA: Ribonucleic acid
WES: Whole exome sequencing
WGS: Whole genome sequencing
XL: X-linked
