# Supplementary material for: Wide diagnostic and genotypic spectrum in patients with suspected mitochondrial disease
Source: Orphanet J Rare Dis. 2023 Oct 2;18:307. doi: 10.1186/s13023-023-02921-0 (PMC10544509; doi:10.1186/s13023-023-02921-0)
Supplement: Supplementary file 1 — Additional file 1: Table S1. Oligonucleotide sequences for PCR amplification of mtDNA. Table S2. Oligonucleotide sequencing primers for mtDNA Sanger sequencing. Table S3. A list of 751 targeted genes associated with mitochondrial and other neuromuscular disorders tested using targeted gene next generation sequencing technology (Ion AmpliSeq™ Neurological Research Panel). MD-associated genes (99) are bolded [file 13023_2023_2921_MOESM1_ESM.docx]

Supplementary data

Table S1. Oligonucleotide sequences for PCR amplification of mtDNA.

| No. | Oligonucleotide primer name | Oligonucleotide primer sequence | PCR amplicon | PGR product length (bp) |
| --- | --- | --- | --- | --- |
| 1. | mtDNA_14898_F1-1 | 5’-TAGCCATGCACTACTCACCAGA-3’ | 1-1  (14898-151) | 1822 |
| 2. | mtDNA_151_R1-1 | 5’-GGATGAGGCAGGAATCAAAGAC-3’ |  |  |
| 3. | mtDNA_16488_F2-1 | 5’-CTGTATCCGACATCTGGTTCCT-3’ | 2-1  (16488-1677) | 1758 |
| 4. | mtDNA_1677_R2-1 | 5’-GTTTAGCTCAGAGCGGTCAAGT-3’ |  |  |
| 5. | mtDNA_1404_F3-1 | 5’-ACTTAAGGGTCGAAGGTGGATT-3’ | 3-1  (1404-3947) | 2543 |
| 6. | mtDNA_3947_R3-1 | 5’-TCGATGTTGAAGCCTGAGACTA-3’ |  |  |
| 7. | mtDNA_3734_F4-1 | 5’-AAGTCACCCTAGCCATCATTCTA-3’ | 4-1  (3734-6739) | 3005 |
| 8. | mtDNA_6739_R4-1 | 5’-GATATCATAGCTCAGACCATACC-3’ |  |  |
| 9. | mtDNA_6511_F5-1 | 5’-CTGCTGGCATCACTATACTACTA-3’ | 5-1  (6511-9220) | 2709 |
| 10. | mtDNA_9220_R5-1 | 5’-GATTGGTGGGTCATTATGTGTTG-3’ |  |  |
| 11. | mtDNA_8910_F6-1 | 5’-CTTACCACAAGGCACACCTACA-3’ | 6-1  (8910-10648) | 1738 |
| 12. | mtDNA_10648_R6-1 | 5’-GGCACAATATTGGCTAAGAGGG-3’ |  |  |
| 13. | mtDNA_10360_F7-1 | 5’-GTCTGGCCTATGAGTGACTACA-3’ | 7-1  (10360-12226) | 1866 |
| 14. | mtDNA_12226_R7-1 | 5’-CAGTTCTTGTGAGCTTTCTCGG-3’ |  |  |
| 15. | mtDNA_11977_F8-1 | 5’-CTCCCTCTACATATTTACCACAAC-3’ | 8-1  (11977-13830) | 1853 |
| 16. | mtDNA_13830_R8-1 | 5’-AAGTCCTAGGAAAGTGACAGCGA-3’ |  |  |
| 17. | mtDNA_13477_F9-1 | 5’-GCAGGAATACCTTTCCTCACAG-3’ | 9-1  (13477-15349) | 1872 |
| 18. | mtDNA_15349_R9-1 | 5’-GTGCAAGAATAGGAGGTGGAGT-3’ |  |  |

Table S2. Oligonucleotide sequencing primers for mtDNA Sanger sequencing.

| No. | Oligonucleotide primer name | Oligonucleotide primer sequence | PCR amplicon |
| --- | --- | --- | --- |
| 1. | mtDNA_14898_F1-1 | 5’-TAGCCATGCACTACTCACCAGA-3’ | 1-1  (14898-151) |
| 2. | mtDNA_151_R1-1 | 5’-GGATGAGGCAGGAATCAAAGAC-3’ |  |
| 3. | mtDNA_15416_F1-2 | 5’-TACACAATCAAAGACGCCCTC-3’ |  |
| 4. | mtDNA_16281_R1-2 | 5’-GTTGGTATCCTAGTGGGTGAG-3’ |  |
| 5. | mtDNA_15966_F1-3 | 5’-AGTCTTTAACTCCACCATTAG-3’ |  |
| 6. | mtDNA_15825_R1-3 | 5’-GTGAAGTATAGTACGGATGCT-3’ |  |
| 7. | mtDNA_16488_F2-1 | 5’-CTGTATCCGACATCTGGTTCCT-3’ | 2-1  (16488-1677) |
| 8. | mtDNA_1677_R2-1 | 5’-GTTTAGCTCAGAGCGGTCAAGT-3’ |  |
| 9. | mtDNA_411_F2-2 | 5’-CGGTATGCACTTTTAACAGTC-3’ |  |
| 10. | mtDNA_1159_R2-2 | 5’-TAAGCTGTGGCTCGTAGTGT-3’ |  |
| 11. | mtDNA_909_F2-3 | 5’-GATTAACCCAAGTCAATAGAA-3’ |  |
| 12. | mtDNA_638_R2-3 | 5’-GGTGATGTGAGCCCGTCTAAA-3’ |  |
| 13. | mtDNA_1404_F3-1 | 5’-ACTTAAGGGTCGAAGGTGGATT-3’ | 3-1  (1404-3947) |
| 14. | mtDNA_3947_R3-1 | 5’-TCGATGTTGAAGCCTGAGACTA-3’ |  |
| 15. | mtDNA_2028_F3-2 | 5’-GATAGAATCTTAGTTCAACTT-3’ |  |
| 16. | mtDNA_3382_R3-2 | 5’-TTCGTTCGGTAAGCATTAGGA-3’ |  |
| 17. | mtDNA_2646_F3-3 | 5’-GGTTCAGCTGTCTCTTACTTT-3’ |  |
| 18. | mtDNA_2801_R3-3 | 5’-TAATGCAGGTTTGGTAGTTTA-3’ |  |
| 19. | mtDNA_3239_F3-4 | 5’-GCAGAGCCCGGTAATCGCATA-3’ |  |
| 20. | mtDNA_2263_R3-4 | 5’-GGGTGTGAGGAGTTCAGTTAT-3’ |  |

Table S2. (continued)

| No. | Oligonucleotide primer name | Oligonucleotide primer sequence | PCR amplicon |
| --- | --- | --- | --- |
| 21. | mtDNA_3734_F4-1 | 5’-AAGTCACCCTAGCCATCATTCTA-3’ | 4-1  (3734-6739) |
| 22. | mtDNA_6739_R4-1 | 5’-GATATCATAGCTCAGACCATACC-3’ |  |
| 23. | mtDNA_4346_F4-2 | 5’-GAACCCATCCCTGAGAATCCA-3’ |  |
| 24. | mtDNA_6154_R4-2 | 5’-GGAACTAGTCAGTTGCCAAAG-3’ |  |
| 25. | mtDNA_4896_F4-3 | 5’-TACCAAATCTCTCCCTCACTA-3’ |  |
| 26. | mtDNA_5571_R4-3 | 5’-AAGTATTGCAACTTACTGAGG-3’ |  |
| 27. | mtDNA_5468_F4-4 | 5’-CACGCTACTCCTACCTATCTC-3’ |  |
| 28. | mtDNA_5017_R4-4 | 5’-GAGGAGTATGCTAAGATTTTG-3’ |  |
| 29. | mtDNA_5995_F4-5 | 5’-CAGCTCTAAGCCTCCTTATTC-3’ |  |
| 30. | mtDNA_4421_R4-5 | 5’-CTTATTTAGCTGACCTTACTT-3’ |  |
| 31. | mtDNA_6511_F5-1 | 5’-CTGCTGGCATCACTATACTACTA-3’ | 5-1  (6511-9220) |
| 32. | mtDNA_9220_R5-1 | 5’-GATTGGTGGGTCATTATGTGTTG-3’ |  |
| 33. | mtDNA_7111_F5-2 | 5’-ACACCCTAGACCAAACCTACG-3’ |  |
| 34. | mtDNA_8600_R5-2 | 5’-AGAATGATCAGTACTGCGGCG-3’ |  |
| 35. | mtDNA_7713_F5-3 | 5’-TCCTAACACTCACAACAAAAC-3’ |  |
| 36. | mtDNA_8000_R5-3 | 5’-CAACGTCAAGGAGTCGCAGGT-3’ |  |
| 37. | mtDNA_8311_F5-4 | 5’-TAGCATTAACCTTTTAAGTTA-3’ |  |
| 38. | mtDNA_7359_R5-4 | 5’-CTACTATTAGGACTTTTCGCT-3’ |  |
| 39. | mtDNA_8910_F6-1 | 5’-CTTACCACAAGGCACACCTACA-3’ | 6-1  (8910-10648) |
| 40. | mtDNA_10648_R6-1 | 5’-GGCACAATATTGGCTAAGAGGG-3’ |  |
| 41. | mtDNA_9393_F6-2 | 5’-CGAGAAAGCACATACCAAGGC-3’ |  |
| 42. | mtDNA_10154_R6-2 | 5’-TTCTATGTAGCCGTTGAGTTG-3’ |  |
| 43. | mtDNA_9874_F6-3 | 5’-TAATATTTCACTTTACATCCA-3’ |  |
| 44. | mtDNA_9647_R6-3 | 5’-AGCTCAGGTGATTGATACTCC-3’ |  |
| 45. | mtDNA_10360_F7-1 | 5’-GTCTGGCCTATGAGTGACTACA-3’ | 7-1  (10360-12226) |
| 46. | mtDNA_12226_R7-1 | 5’-CAGTTCTTGTGAGCTTTCTCGG-3’ |  |
| 47. | mtDNA_10892_F7-2 | 5’-ATCAACAACAACCTATTTAGC-3’ |  |
| 48. | mtDNA_11673_R7-2 | 5’-GTTTGGATGAGAATGGCTGTT-3’ |  |
| 49. | mtDNA_11461_F7-3 | 5’-ACTCTTAAAACTAGGCGGCTA-3’ |  |
| 50. | mtDNA_11163_R7-3 | 5’-CGGGTGATGATAGCCAAGGTG-3’ |  |
| 51. | mtDNA_11977_F8-1 | 5’-CTCCCTCTACATATTTACCACAAC-3’ | 8-1  (11977-13830) |
| 52. | mtDNA_13830_R8-1 | 5’-AAGTCCTAGGAAAGTGACAGCGA-3’ |  |
| 53. | mtDNA_12500_F8-2 | 5’-TGTGCCTAGACCAAGAAGTTA-3’ |  |
| 54. | mtDNA_13297_R8-2 | 5’-GGTTGATGCCGATTGTAACTA-3’ |  |
| 55. | mtDNA_12988_F8-3 | 5’-CTAGCAGCAGCAGGCAAATCA-3’ |  |
| 56. | mtDNA_12763_R8-3 | 5’-CGATGAACAGTTGGAATAGGT-3’ |  |
| 57. | mtDNA_13477_F9-1 | 5’-GCAGGAATACCTTTCCTCACAG-3’ | 9-1  (13477-15349) |
| 58. | mtDNA_15349_R9-1 | 5’-GTGCAAGAATAGGAGGTGGAGT-3’ |  |
| 59. | mtDNA_13950_F9-2 | 5’-CTATCTAGGCCTTCTTACGAG-3’ |  |
| 60. | mtDNA_14838_R9-2 | 5’-CATCATGCGGAGATGTTGGAT-3’ |  |
| 61. | mtDNA_14440_F9-3 | 5’-ATACTCCTCAATAGCCATCGC-3’ |  |
| 62. | mtDNA_14325_R9-3 | 5’-AACTTTAATAGTGTAGGAAGC-3’ |  |

Table S3. A list of 751 targeted genes associated with mitochondrial and other neuromuscular disorders tested using targeted gene next generation sequencing technology (Ion AmpliSeq™ Neurological Research Panel). MD-associated genes (99) are bolded.

| **GENES (1/2)** | | | | | | | | |
| --- | --- | --- | --- | --- | --- | --- | --- | --- |
| *AAAS* | *ARX* | *CAV3* | ***COQ2*** | ***EARS2*** | *GABRD* | *HSD17B4* | *LARGE* | ***MTPAP*** |
| *AARS* | *ASCC3* | *CC2D1A* | ***COQ5*** | *EEF1B2* | *GABRG2* | *HSPB1* | *LDB3* | *MUSK* |
| ***AARS2*** | *ASCL1* | *CCDC78* | ***COQ6*** | *EFHC1* | *GAD1* | *HSPB3* | *LGI1* | *NAGA* |
| ***ABAT*** | *ASPM* | *CCDC88C* | ***COX10*** | *EGR2* | *GALC* | *HSPB8* | *LINS* | *NBN* |
| *ABCD1* | *ATCAY* | *CCNA2* | ***COX14*** | *EIF2B1* | *GAMT* | ***HSPD1*** | *LITAF* | *NDE1* |
| *ABHD12* | *ATL1* | *CCT5* | ***COX15*** | *EIF2B2* | *GAN* | ***HTRA2*** | *LMNA* | *NDRG1* |
| ***ACAD9*** | *ATN1* | *CDC6* | ***COX6B1*** | *EIF2B3* | ***GARS*** | ***HTT*** | *LMNB1* | *NDST1* |
| *ACY1* | *ATP13A2* | *CDH15* | *CP* | *EIF2B4* | *GATAD2B* | *HUWE1* | ***LRPPRC*** | ***NDUFA1*** |
| ***ACO2*** | *ATP1A2* | *CDK5RAP2* | *CPA6* | *EIF2B5* | *GATM* | *IAPP* | *LRRK2* | ***NDUFA10*** |
| *ACSL4* | *ATP1A3* | *CDKL5* | *CRADD* | *EIF4G1* | *GBA2* | *YARS* | *LRSAM1* | ***NDUFA11*** |
| *ACTA1* | *ATP2A1* | *CDON* | *CRBN* | *ELP2* | ***GDAP1*** | *IER3IP1* | *MAGT1* | ***NDUFA12*** |
| *ACTB* | *ATP2B3* | *CENPJ* | *CRYAB* | *EMD* | *GDI1* | *IGBP1* | *MAN1B1* | ***NDUFA2*** |
| *ACTG1* | ***ATP5E*** | *CEP135* | *CSF1R* | *EMX2* | *GFAP* | *IGHMBP2* | *MAOA* | *NDUFA7* |
| *ADAR* | *ATP6AP2* | *CEP152* | *CSNK1D* | *ENTPD1* | ***GFER*** | *IKBKAP* | *MAPK10* | ***NDUFA9*** |
| ***ADCK3*** | *ATP7A* | *CEP41* | *CSTB* | *EPB41L1* | *GFPT1* | *IL1RAPL1* | *MAPT* | *NDUFAB1* |
| *ADK* | ***ATPAF2*** | *CEP57* | *CTNNB1* | *EPM2A* | *GIGYF2* | *INF2* | *MATR3* | ***NDUFAF1*** |
| *ADRA2B* | *ATR* | *CEP63* | *CTSD* | *ERBB3* | *GJB1* | *INPP4A* | *MBD5* | ***NDUFAF2*** |
| *ADSL* | *ATRX* | *CFL2* | *CUL4B* | *ERLIN2* | *GJC2* | *INPP5E* | *MCPH1* | ***NDUFAF3*** |
| *AFF2* | *ATXN1* | *CHAT* | *D4Z4* | *EXOSC3* | *GLI2* | *IQSEC2* | *MECP2* | ***NDUFAF4*** |
| ***AFG3L2*** | *ATXN10* | ***CHKB*** | *DAG1* | *FA2H* | *GLRA1* | ***ISCU*** | *MED12* | ***NDUFAF5*** |
| *AGRN* | *ATXN2* | *CHMP1A* | ***DARS2*** | *FAM126A* | *GLRB* | *ISPD* | *MED13L* | ***NDUFAF6*** |
| *AGTR2* | *ATXN3* | *CHMP2B* | *DCC* | *FAM134B* | *GNAL* | *ITGA7* | *MED17* | ***NDUFB3*** |
| *AHI1* | *ATXN7* | *CHRNA1* | *DCTN1* | ***FARS2*** | *GNB4* | *ITM2B* | *MED23* | ***NDUFS1*** |
| *AIMP1* | *ATXN8OS* | *CHRNA2* | *DCX* | *FASN* | *GNE* | *ITPR1* | *MED25* | ***NDUFS2*** |
| *ALDH5A1* | *AUTS2* | *CHRNA4* | *DDHD1* | ***FASTKD2*** | *GON4L* | *JPH3* | ***MEF2C*** | ***NDUFS3*** |
| *ALDH7A1* | *B3GALNT2* | *CHRNB1* | *DDHD2* | *FBXO7* | *GOSR2* | *KANK1* | *MEGF10* | ***NDUFS4*** |
| *ALS2* | *BAG3* | *CHRNB2* | *DES* | *FGD1* | *GPHN* | ***KARS*** | ***MFF*** | *NDUFS5* |
| *AMPD1* | ***BCS1L*** | *CHRND* | *DHTKD1* | *FGD4* | *GPR56* | *KBTBD13* | *MFSD8* | ***NDUFS6*** |
| *ANG* | *BDNF* | *CHRNE* | *DYNC1H1* | *FGF14* | *GPR98* | *KCNA1* | *MYBPC1* | ***NDUFS7*** |
| *ANO10* | *BIN1* | *CHRNG* | *DIP2B* | *FHL1* | *GRIA3* | *KCNC3* | *MYF6* | ***NDUFS8*** |
| *ANO3* | *BRAT1* | *CYP27A1* | *DYRK1A* | *FIG4* | *GRIK2* | *KCNJ10* | *MYH2* | ***NDUFV1*** |
| *ANO5* | *BRWD3* | *CYP2U1* | *DIS3L2* | *FKRP* | *GRIN1* | *KCNMA1* | *MYH3* | ***NDUFV2*** |
| *AP1S2* | *BSCL2* | *CYP7B1* | *DYSF* | *FKTN* | *GRIN2A* | *KCNQ2* | *MYH7* | *NEB* |
| *AP4B1* | *BUB1B* | *CLCN1* | *DLG3* | *FLNA* | *GRIN2B* | *KCNQ3* | *MYH8* | *NEFL* |
| *AP4E1* | *C12orf57* | *CLCN2* | *DMD* | *FLNC* | *GRM1* | *KCNT1* | *MYOT* | ***NFU1*** |
| *AP4M1* | ***C12orf65*** | *CLN3* | *DMPK* | *FLVCR2* | *GRN* | *KCTD7* | *MLC1* | *NGF* |
| *AP4S1* | ***C19orf12*** | *CLN5* | ***DNA2*** | *FMR1* | *GTDC2* | *KDM5A* | *MMD2* | *NHEJ1* |
| *AP5Z1* | *C5orf42* | *CLN6* | *DNAJB2* | *FOLR1* | ***HCFC1*** | *KDM5C* | *MOG* | *NHLRC1* |
| *APOB* | *C9orf72* | *CLN8* | *DNAJB6* | *FOXG1* | *HDAC1* | *KDM6B* | ***MPC1*** | *NIN* |
| *APTX* | *CA8* | *CNBP* | *DNAJC5* | *FOXP1* | *HDAC4* | *KIAA0196* | *MPDZ* | *NIPA1* |
| *AR* | *CACNA1A* | *CNKSR1* | *DNM2* | *FOXP2* | *HEPACAM* | *KIF1A* | *MPP3* | *NKX2-1* |
| *ARFGEF2* | *CACNA1G* | *CNTN1* | *DNMT1* | ***FOXRED1*** | *HEXA* | *KIF1B* | *MPZ* | *NLGN3* |
| *ARHGEF6* | *CACNB4* | *CNTNAP2* | *DOK7* | *FRY* | *HYLS1* | *KIF5A* | *MR1* | *NLGN4X* |
| *ARHGEF9* | *CACNG2* | ***COA5*** | *DPAGT1* | *FTL* | *HINT1* | *KIF7* | *MRE11A* | *NOL3* |
| *ARID1A* | *CAMTA1* | *COG6* | *DPYD* | *FTSJ1* | *HIST1H4B* | *KIRREL3* | *MRI1* | *NOP56* |
| *ARID1B* | *CAPN10* | *COL4A2* | *DPM2* | *FUS* | *HIST3H3* | *L1CAM* | ***MRPL3*** | *NPC1* |
| *ARL13B* | *CAPN3* | *COL6A1* | *DRD2* | *FUZ* | *HK1* | *LAMA1* | *MSTN* | *NPC2* |
| *ARL14EP* | *CASC5* | *COL6A2* | *DRD4* | ***FXN*** | *HOXB1* | *LAMA2* | ***MTFMT*** | *NRXN1* |
| *ARNT2* | *CASK* | *COL6A3* | *DRD5* | *GABRA1* | *HOXD10* | *LAMB1* | *MTM1* | *NTRK1* |
| *ARSA* | *CASP2* | *COLQ* | *DST* | *GABRB3* | ***HSD17B10*** | *LAMC3* | *MTMR2* | ***NUBPL*** |

Table S3. (continued)

| **GENES (2/2)** | | | | | | | |
| --- | --- | --- | --- | --- | --- | --- | --- |
| *NUP62* | *PHYH* | *PSAP* | *SCN1A* | *SLC25A22* | *ST3GAL5* | *TNNT1* | *UBR7* |
| *OCLN* | *PHOX2B* | *PSEN1* | *SCN1B* | *SLC2A1* | *STAMBP* | *TNNT3* | *UPF3B* |
| *OCRL* | *PIGN* | *PSEN2* | *SCN2A* | *SLC30A10* | *STIL* | *TOR1A* | ***UQCRB*** |
| *OPHN1* | *PIK3R2* | *PTCH1* | *SCN4A* | *SLC31A1* | *STIM1* | ***TPK1*** | ***UQCRC2*** |
| *OPTN* | *PIK3R5* | *PTEN* | *SCN8A* | *SLC33A1* | *STRADA* | *TPM2* | ***UQCRQ*** |
| *ORC1* | *PINK1* | *RAB18* | *SCN9A* | *SLC35A2* | *STXBP1* | *TPM3* | *UROC1* |
| *ORC6* | *PIP5K1C* | *RAB39B* | ***SCO1*** | *SLC52A2* | ***SUCLG1*** | *TPP1* | *VANGL1* |
| *PABPN1* | *PLA2G6* | *RAB7A* | ***SCO2*** | *SLC52A3* | *SUOX* | *TRAPPC9* | *VAPB* |
| *PACS1* | *PLCB1* | *RABL6* | ***SDHA*** | *SLC5A7* | ***SURF1*** | *TREM2* | *VCP* |
| *PAFAH1B1* | *PLEC* | *RAD50* | ***SDHAF1*** | *SLC6A3* | ***TACO1*** | *TREX1* | *VLDLR* |
| *PAK3* | *PLEKHG5* | *RAI1* | *SEPN1* | *SLC6A5* | *TAF1* | *TRIM32* | *VMA21* |
| ***PANK2*** | *PLP1* | *RALGDS* | *SEPSECS* | *SLC6A8* | *TAF2* | *TRMT1* | *VPS13A* |
| *PARK2* | *PMM2* | *RAPSN* | *SEPT9* | *SLC9A6* | *TARDBP* | *TRPA1* | *VPS33B* |
| *PARK7* | *PMP22* | ***RARS2*** | *SERPINI1* | *SLITRK1* | *TBC1D24* | *TRPV4* | *VPS35* |
| *PARP1* | *PNKD* | *RBBP8* | *SETX* | *SMARCA2* | *TBP* | *TSC1* | *VPS37A* |
| ***PC*** | *PNKP* | *REEP1* | *SGCA* | *SMARCA4* | *TCAP* | *TSC2* | *VRK1* |
| *PCDH19* | *PNPLA2* | *RELN* | *SGCB* | *SMARCB1* | *TCTN1* | *TSEN2* | *WDR45* |
| *PDGFRB* | *PNPLA6* | *RGS7* | *SGCD* | *SMCHD1* | *TDGF1* | *TSEN34* | *WDR45B* |
| ***PDHA1*** | *PNPO* | *RYR1* | *SGCE* | *SMN1* | *TDP1* | *TSEN54* | *WDR62* |
| ***PDHB*** | ***POLG*** | ***RMND1*** | *SGCG* | *SMS* | *TECPR2* | ***TSFM*** | *WDR81* |
| ***PDHX*** | *POLR3A* | *RNASEH2A* | *SH3TC2* | *SNAP29* | *TECR* | *TSPAN7* | *WNK1* |
| *PDYN* | *POLR3B* | *RNASEH2B* | *SHANK2* | *SNCA* | *TFG* | *TTBK2* | *ZBTB40* |
| ***PDSS2*** | *POMGNT1* | *RNASEH2C* | *SHANK3* | *SNCB* | *TGIF1* | ***TTC19*** | *ZCCHC8* |
| *PDX1* | *POMT1* | *RNASET2* | *SHH* | *SNIP1* | *TGM6* | *TTI2* | *ZDHHC15* |
| *PECR* | *POMT2* | *RNF170* | *SHROOM4* | *SOBP* | *TH* | *TTN* | *ZDHHC9* |
| *PEX1* | *PPP2R2B* | *RNU4ATAC* | *SIGMAR1* | *SOD1* | *THAP1* | *TTPA* | *ZEB2* |
| *PEX12* | *PPT1* | *ROGDI* | *SIL1* | *SPAST* | *TIA1* | *TUBA1A* | *ZFYVE26* |
| *PEX14* | *PQBP1* | *RPGRIP1L* | *SYN1* | *SPG11* | ***TYMP*** | *TUBA8* | *ZFYVE27* |
| *PEX16* | *PRICKLE1* | *RPS6KA3* | *SYNE1* | *SPG20* | *TYROBP* | *TUBB2B* | *ZIC2* |
| *PEX2* | *PRICKLE2* | ***RRM2B*** | *SYNE2* | *SPG21* | ***TK2*** | *TUBB3* | *ZNF335* |
| *PEX26* | *PRKCG* | *RTN2* | *SYNGAP1* | ***SPG7*** | *TMEM135* | *TUBB4A* | *ZNF41* |
| *PEX3* | *PRKRA* | *RTTN* | *SYP* | *SPR* | *TMEM138* | *TUBGCP6* | *ZNF526* |
| *PEX5* | *PRMT10* | ***SACS*** | *SYT14* | *SPTAN1* | *TMEM216* | ***TUFM*** | *ZNF592* |
| *PEX6* | *PRNP* | ***SAMHD1*** | *SIX3* | *SPTBN2* | *TMEM237* | *TUSC3* | *ZNF674* |
| *PFN1* | *PRPS1* | *SBF2* | *SLC12A6* | *SPTLC1* | *TMEM5* | *UBA1* | *ZNF711* |
| *PHC1* | *PRRT2* | *SC5D* | *SLC1A3* | *SPTLC2* | *TMEM67* | *UBE2A* | *ZNF81* |
| *PHF8* | *PRSS12* | *SCAPER* | *SLC20A2* | *SRPX2* | ***TMEM70*** | *UBE3A* |  |
| *PHGDH* | *PRX* | *SCARB2* | ***SLC25A19*** | *ST3GAL3* | *TNNI2* | *UBQLN2* |  |
